# Supplementary material for: SPEADI: Accelerated Analysis of IDP-Ion Interactions from MD-Trajectories
Source: Biology (Basel). 2023 Apr 10;12(4):581. doi: 10.3390/biology12040581 (PMC10135740; doi:10.3390/biology12040581)
Supplement: Supplementary file 1 [file biology-12-00581-s001.zip › biology-2320538-supplementary.pdf]

Supplementary Information for SPEADI: Accelerated Analysis of IDP-Ion Interactions From MD-Trajectories

Emile de Bruyn[1,2], Anton Emil Dorn[2], Olav Zimmermann[1], and Giulia Rossetti[1,3,4]

[1] *Jülich Supercomputing Centre, Forschungszentrum Jülich, 52425 Jülich, Germany*
[2] *Faculty of Mathematics, Computer Science and Natural Sciences, RWTH Aachen University, 52062 Aachen, Germany*
[3] *Computational Biomedicine, Institute for Advanced Simulation IAS-5 and Institute of Neuroscience and Medicine INM-9, Forschungszentrum Jülich, 52425 Jülich, Germany*
[4] *Department of Neurology, RWTH Aachen University, 52062 Aachen, Germany*

1 VAN HOVE CORRELATION FUNCTIONS

The van Hove Function (VHF) $(G(r,\tau))$ is often used in Molecular Dynamics (MD) simulations of liquids, gasses, and solid materials where it quantifies the stability of local structures and thus the interaction between molecules. $G(r,\tau)$ may be determined experimentally using quasi-elastic and inelastic scattering of both X-ray or neutron radiation using its relation to the Dynamic Scattering factor. [1;2] Shinohara et al. have demonstrated the ability of simulations to correctly identify correlated motions in liquids. [3] We attempted to apply these to ion clouds surrounding biomolecules, but found that the concept of the Time-Resolved Radial Distribution Function (TRRDF) is better suited to simulations of freely moving single proteins using periodic boudary conditions. Nevertheless, SPEADI provides and efficient implementation of the VHF that might be of interest to the Physical Chemistry community.

The VHF is a dynamic correlation function that quantifies the correlation of two particle types in both temporal and spatial dimensions. [4] The VHF may generally be defined as the bivariate distribution over distance r and time τ,

$$G_{a,b}(r,\tau) = \frac{1}{V(r)\rho_b^{\text{bulk}} N_a} \sum_{i\in a}^{N_a} \sum_{j\in b}^{N_b} \delta\left(r - |x_i(0) - x_j(\tau)|\right), \tag{1}$$

between particles of type a and b, with N_a and N_b being the number of particles of type a and b. r represents the value of the distance bin, and $r_i(0)$ and $r_j(\tau)$ represent the position coordinates of particle i at time $\tau = 0$ and particle j at the time being sampled. As the peaks in the distribution quickly dissipate, time τ is only meaningful on a very short time-scale dependent on the particle rate of diffusion. The function is instead sampled at different starting times $t = \tau_0$ and an average over the displacement or relaxation times τ with respect to τ_0 is calculated.

The VHF is normalized by the volume of the radial shell $V(r)$:

$$V(r) = \int_{r_{\text{inner}}}^{r_{\text{outer}}} 4\pi r^2 \,\mathrm{d}r = \frac{4}{3}\pi\left(r_{\text{end}}^3 - r_{\text{start}}^3\right). \tag{2}$$

When the VHF is calculated between particles of the same type, $G(r,\tau)$ may be split into a *self* $G_s(r,\tau)$ and *distinct* part $G_d(r,\tau)$:

$$G(r,\tau) = G_s(r,\tau) + G_d(r,\tau) \tag{3}$$

$$G_s(r,\tau) = \frac{1}{V(r)\rho_a^{\text{bulk}} N_a} \sum_{i\in a}^{N_a} \delta\left(r - |x_i(0) - x_i(\tau)|\right) \tag{4}$$

$$G_d(r,\tau) = \frac{1}{V(r)\rho_b^{\text{bulk}} N_a} \sum_{i\in a}^{N_a} \sum_{j\in b}^{N_b} \delta\left(r - |x_i(0) - x_j(\tau)|\right). \tag{5}$$

The self part of the VHF is related to the particle diffusion.[3;5–8] Integration of $G_s(r,\tau)$ gives the fraction of particles having moved less than a distance threshold within a given time.[1]

Note that the Radial Distribution Function (RDF) $g(r)$ is readily described as a special case of the VHF at relaxation time $\tau = 0$:

$$G_{a,b}(r,\tau=0) = \frac{1}{V(r)\rho_b^{\text{bulk}} N_a} \sum_{i\in a}^{N_a} \sum_{j\in b}^{N_b} \delta(r - |x_i(0) - x_j(0)|), \tag{6}$$

which simplifies to

$$g_{a,b}(r) = \frac{1}{V(r)\rho_b^{\text{bulk}} N_a} \sum_{i\in a}^{N_a} \sum_{j\in b}^{N_b} \delta(r - |x_i - x_j|). \tag{7}$$

VHFs are calculated by constructing a time-distance matrix between the target group (using only the positions in the first frame of the window) and the reference group (using all frames in the window). A histogram is constructed for each of the frames (displacement time with respect to the first frame) in the window.

2 SPEADI DEPENDENCIES AND USAGE

2.1 *Dependencies*

SPEADI is implemented on the basis of, and thus has a hard dependency on, the Python package MDTraj.[9] MDTraj provides low-level functions for reading trajectories and topologies from atomistic simulations of various formats, as well as many analysis methods itself. It also provides optimized methods of iterating over simulation trajectories in many formats, from a large number of simulation programs and codes. Accordingly, SPEADI has followed the naming conventions and general style of MDTraj, so as to smoothly integrate usage of both. The minimum requirements for SPEADI are Python 3.8 and MDTraj 1.5.0.

2.2 *Optional Dependencies*

SPEADI can take advantage of several optional acceleration libraries in the Python environment into which it is installed. The JAX library[10] and its companion XLA compiler provide acceleration on both CPUs, GPUs and Google Cloud-TPUs. It is currently the acceleration library used in AlphaFold2[11;12] and the TensorFlow[13] package. JAX is thus already installed and available in many High Performance Computing (HPC) environments as well as Cloud Computing services

such as Google Co-Lab. SPEADI automatically detects an installation of JAX, and uses it to *just-in-time* (jit) compile performance critical functions for any available accelerators.

SPEADI may also be accelerated with the Python package Numba.[14] This is done by detecting the presence of the Numba package in the Python environment that SPEADI is installed in. Performance-relevant low level functions are jit-compiled and parallelized using the provisions provided by Numba.

When neither JAX nor Numba are detected, SPEADI falls back on a vectorized approach using Numpy arrays[15;16] and MDTraj's (CPU optimized) time-distance matrix function. It is highly recommended to use an accelerated version of the package for either any trajectory with added temporal resolution (i.e. frames saved frequently) or when analysis involves a large number of particles. SPEADI only takes advantage of the shared memory parallelism in both acceleration libraries, as reading large Molecular Dynamics (MD) trajectories are currently the prominent bottleneck. See section 7 and table S2 below for further discussion.

2.3 *Usage*

First, groups for the reference and target in the system topology need to be specified as Python list objects using the MDTraj package in a python script or Jupyter Notebook:

```
import mdtraj as md

top = md.load('topology.pdb').top
group1 = [top.select('<selection_expression>')]
group2 = [top.select('<selection_expression>')]
```

For details on the selection expressions, please refer to the MDTraj documentation. For example, to the calculate the Time-Resolved Radial Distribution Function (TRRDF) between Cβ atoms in separate serine residues and water oxygen atoms (using the TIP4P water model), define the groups as following:

```
top = md.load('topology.pdb').top
group1 = [[a] for a in top.select('resname SER and name CB')]
group2 = [top.select('name OW')]
```

The following code calculates the TRRDF between these two groups using SPEADI:

```
import speadi as sp

r, grt = sp.trrdf(traj, group1, group2, top=top,
                  pbc='general',
                  n_windows=2000, window_size=500,
                  stride=1, skip=1,
                  r_range=(0.0, 2.0), nbins=200)
```

Here, specifying `pbc` allows the user to enable acceleration for periodic boundary conditions in cubic simulation cell geometries ("cubic"), where the default "general" option can be used for arbitrary simulation cell geometries such as "dodecahedral" simulation cells. `stride` tells the function to either calculate using every frame, or to only use every *n*th frame in the trajectory.

Obtaining the time-averaged Radial Distribution Function (RDF) is as simple as averaging over the first dimension:

```
gr = np.mean(grt, axis=0)
```

To instead calculate the integral of TRRDF between these two groups:

```
r, nrt = sp.int_trrdf(traj, group1, group2, top=top,
                      pbc='general',
                      n_windows=2000, window_size=500,
                      stride=1, skip=1,
                      r_range=(0.0, 2.0), nbins=200)
```

To calculate the van Hove Function (VHF) for the same trajectory and groups, one simply has to write the following:

```
r, Gs, Gd = sp.vanhove(traj, group1, group2, pbc='general',
                       n_windows=2000, window_size=500,
                       overlap=False,
                       stride=1, skip=1,
                       r_range=(0.0, 2.0), nbins=200)
```

`overlap` specifies either `False`, or an integer number of frames to move the start of a new window forward in time from the start of the previous window. The function returns the variables `r`: the mid-points of each radial slice, G_s: the self-correlation function, and G_d: the distinct part of the van-Hove correlation function averaged over every time window. Each of the variables are returned as Numpy arrays.[15]

Subsection 6.3 contains an example of the VHF applied to ions around a biomolecule.

3 EXPERIMENTAL DETAILS FOR MOLECULAR DYNAMICS SIMULATIONS

3.1 *Alpha-Synuclein*

3.1.1 *Wild-Type Alpha-Synuclein*

A 25 ns all-atom Molecular Dynamics (MD) simulation was conducted for (N-terminally acetylated) wild-type alpha-Synuclein (AS) using the Replica Exchange with Solute Tempering (REST2)-algorithm.[17] 32 replicas between 300 and 500 K exchanged every 100 time steps. The most frequent conformation found during previous simulations by Rossetti et al. was used as the start-

ing structure.[18] The simulation were parameterized using the recent a99SB-*disp* force field[19] in sodium chloride solution at physiological (150 mmol L^{-1}) concentration.

3.1.2 *E46K Alpha-Synuclein*

A 100 ns all-atom MD simulation of AS containing the E46K point mutation associated with early onset Parkinson's Disease (PD) was conducted using the REST2-algorithm.[17] 32 replicas between 300 and 500 K exchanged every 100 time steps. The most frequent conformation found during previous simulations by Rossetti et al. was again used as the starting structure.[18] The simulation was conducted using the recent a99SB-*disp* force field[19] in sodium chloride solution at physiological (150 mmol L^{-1}) concentration.

3.1.3 *Wild-Type Alpha-Synuclein with the DES-Amber Force Field*

A 100 ns all-atom MD simulation of AS containing the E46K point mutation associated with early onset PD was conducted using the REST2-algorithm.[17] 32 replicas between 300 and 500 K exchanged every 100 time steps. The most frequent conformation found during previous simulations by Rossetti et al. was again used as the starting structure.[18] The simulation was conducted using the recent a99SB-*disp* force field[19] in sodium chloride solution at physiological (150 mmol L^{-1}) concentration.

3.2 *Humanin*

1 µs all-atom MD simulations were conducted on wild-type Humanin (HN) and mutants (S14G, D-S14, D-S7 and D-S7,14) using the lowest energy structure of the NMR ensembles deposited in the Protein Data Bank under PDB-code 1Y32.[20] Mutations were done starting from the wild-type structure using PyMOL.[21] The simulations were conducted using the GROMACS[22–27] package (version 2021.4) starting from the HN conformation found from the final frame of the wild-type HN simulation. All simulations were conducted using the a99SB-*disp* all-atom force field specifically designed for Intrinsically Disordered Proteins (IDPs) together with its modified TIP4P-D water model.[19] The simulations were conducted as unbiased MD simulations at 300 K in isothermic-isobaric (NPT) ensembles after a series of equilibration steps. The simulations were performed in sodium chloride solution at physiological (150 mmol L^{-1}) concentration.

A further 500 ns REST2 simulation was performed on the wild-type HN using the same starting equilibrated structure as above. 16 replicas between 300 and 500 K exchanged every 100 time steps.

4 ALPHA-SYNUCLEIN

4.1 *Convergence*

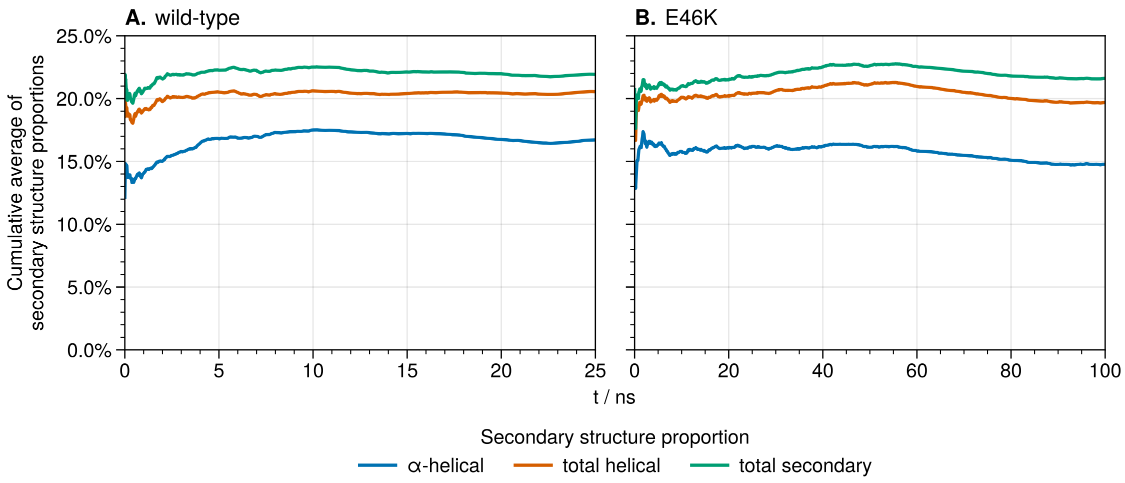

Figure S1: Cumulative average of secondary structure content for the "wet" wild-type (A) and E46K mutated (B) alpha-Synuclein (AS) trajectories.

4.2 *Clustering*

Clustering the simulation trajectories was done according to the recent protocol described by Appadurai et al. for clustering Intrinsically Disordered Protein (IDP) structures.[28] The simulations for wild-type and E46K AS were converged after 12 ns of simulation time. The lowest temperature replica (at 300 K) was used for analysis in both cases. The Root Mean Square Distance (RMSD) was calculated between each frame of the converged part of the trajectories. The wild-type was sampled each 10 ps and the E46K mutant was sampled each 100 ps, resulting in 1300 and 800 frames for clustering respectively.

The RMSD matrix was then clustered using t-distributed Stochastic Neighbor Embedding (t–SNE) at different perplexity values. The perplexity values giving the highest silhouette score was chosen (100 for the wild-type and 350 for the mutant). The t–SNE projection was then clustered using K-means clustering for 20 clusters.

4.3 *Ion Distributions for Each Alpha-Synuclein Domain*

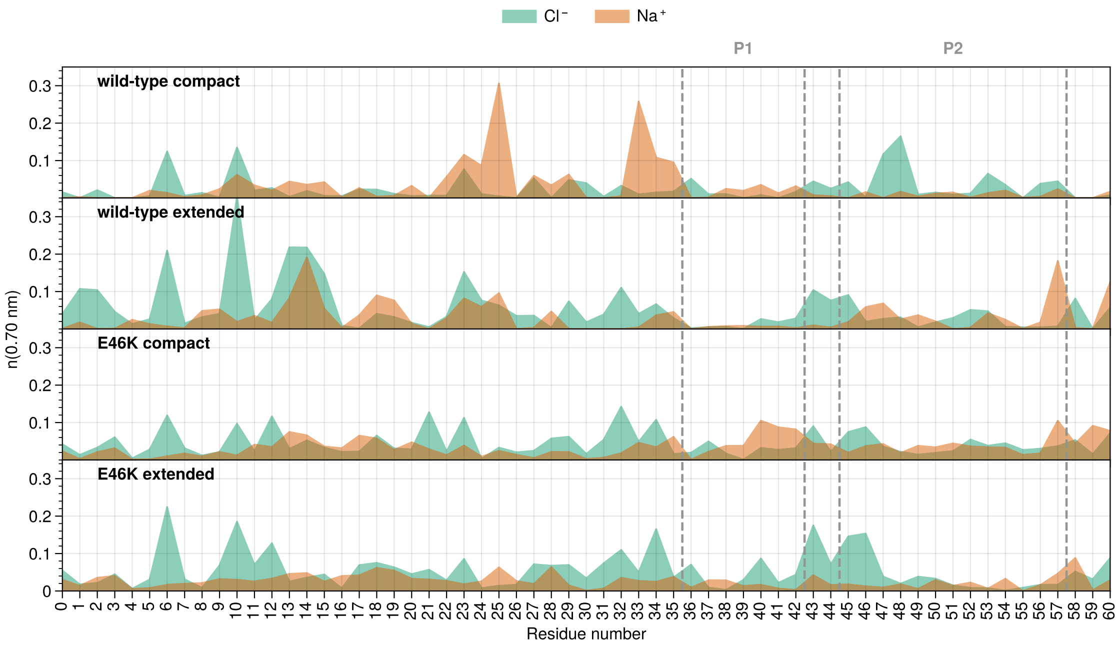

Figure S2: Sodium and chloride ion distributions in the N-terminal region along the residue index for compact and extended conformations of wild-type and E46K-AS. Residue index 0 is the acetylated N-terminus. Mean number of ions (running coordination number), is given as the integral of $g(r)$ for the ion up to the second hydration shell ($r \leq 0.70\,\text{nm}$).

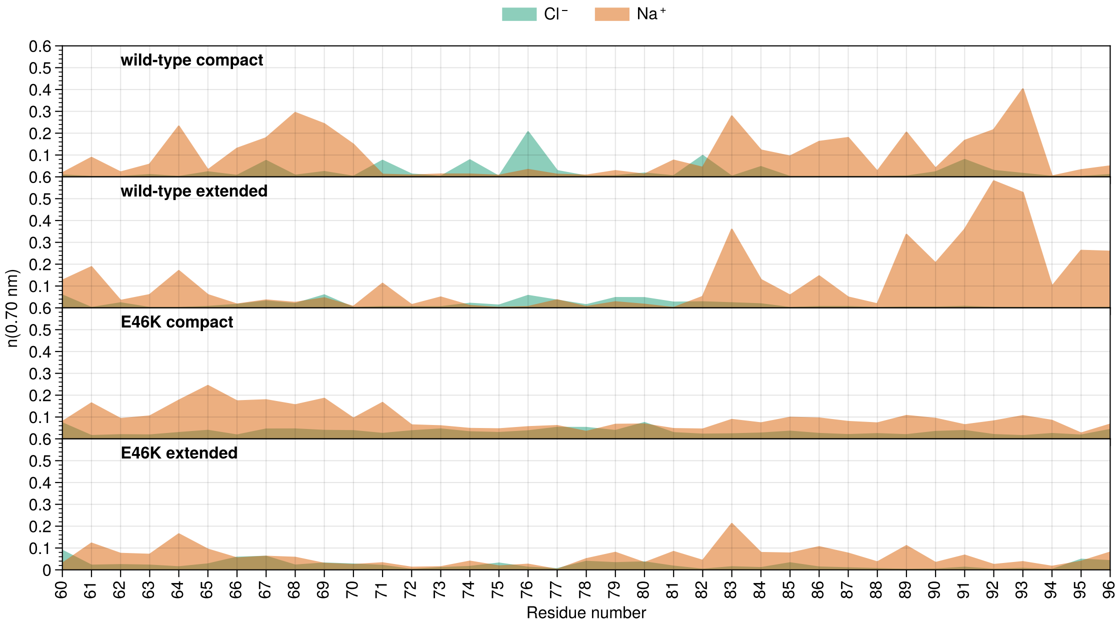

Figure S3: Sodium and chloride ion distributions in the NAC region along the residue index for compact and extended conformations of wild-type and E46K-AS. Mean number of ions (running coordination number), is given as the integral of $g(r)$ for the ion up to the second hydration shell ($r \leq 0.70\,\text{nm}$).

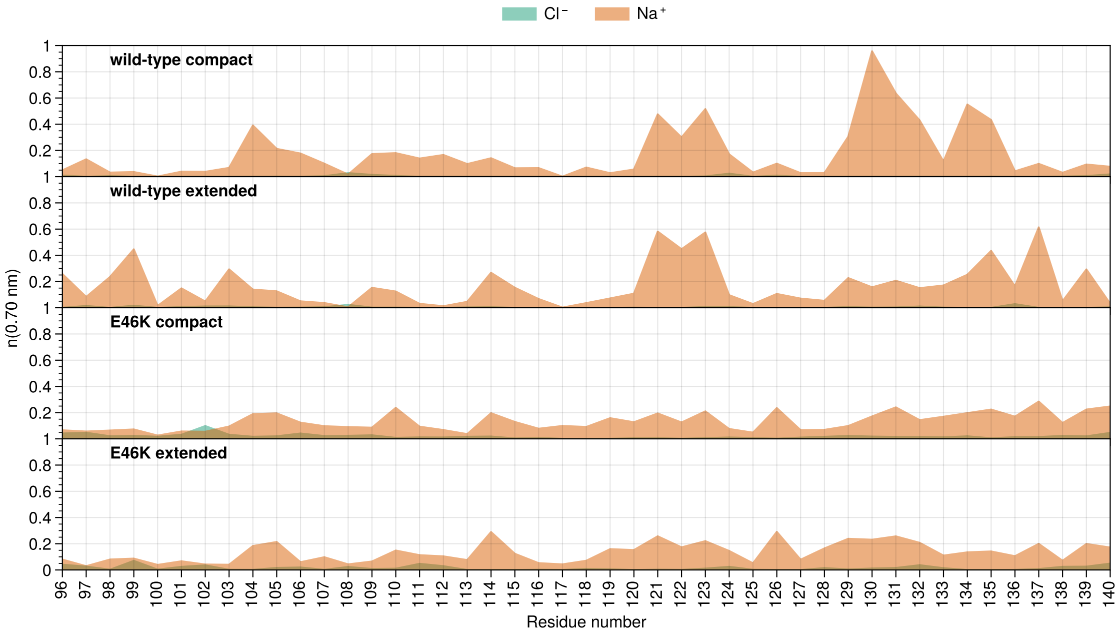

Figure S4: Sodium and chloride ion distributions in the C-terminal region along the residue index for compact and extended conformations of wild-type and E46K-AS. Mean number of ions (running coordination number), is given as the integral of $g(r)$ for the ion up to the second hydration shell ($r \leq 0.70\,\text{nm}$).

4.4 *Choice of Ion Interaction Site*

Ion interaction sites for each amino acid were chosen as the carbon atom two bonds away from a charge carrying atom (table S1).

**Amino acid**	R	H	K	D	E	S	T	N	Q	C	G	P	A	V	I	L	M	F	Y	W
**Carbon atom**	γ	β	δ	α	β	α	α	α	β	α	α	α	β	β	β	β	γ	β	ϵ	β

Table S1: Choice of ion interaction sites for each amino acid.

4.5 *Intradomain Residue Distance Maps of Representative Clusters*

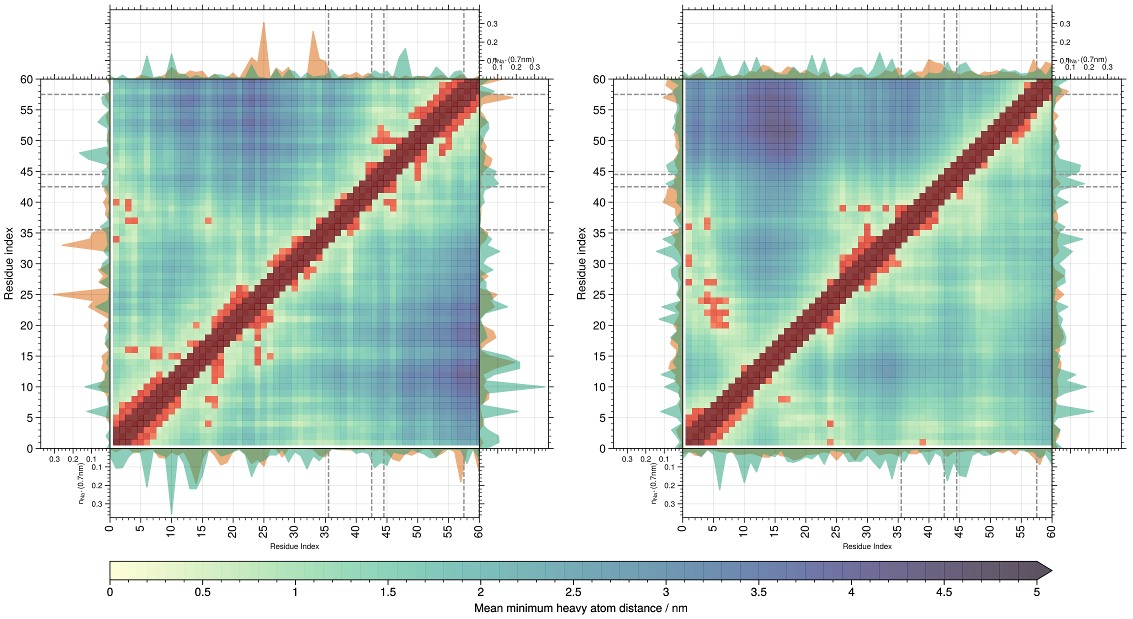

Figure S5: Distance maps between residues 0-59 in representative compact (triangle above) and extended (triangle below) clusters in wild-type (left) and E46K mutated (right) AS. Red points mark contact (mean distance ≤ 0.5 nm). Insets show the mean number of sodium (orange) and chloride (green) ions within a distance of 0.70 nm.

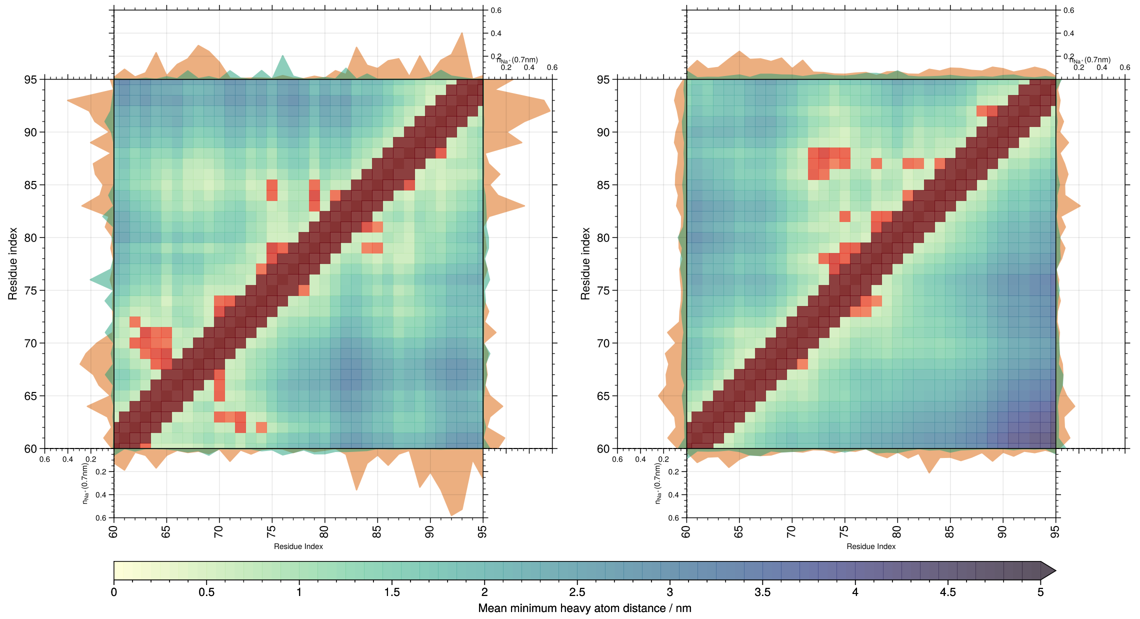

Figure S6: Distance maps between residues 60-96 in representative compact (triangle above) and extended (triangle below) clusters in wild-type (left) and E46K mutated (right) AS. Red points mark contact (mean distance $\leq$ 0.5 nm). Insets show the mean number of sodium (orange) and chloride (green) ions within a distance of 0.70 nm.

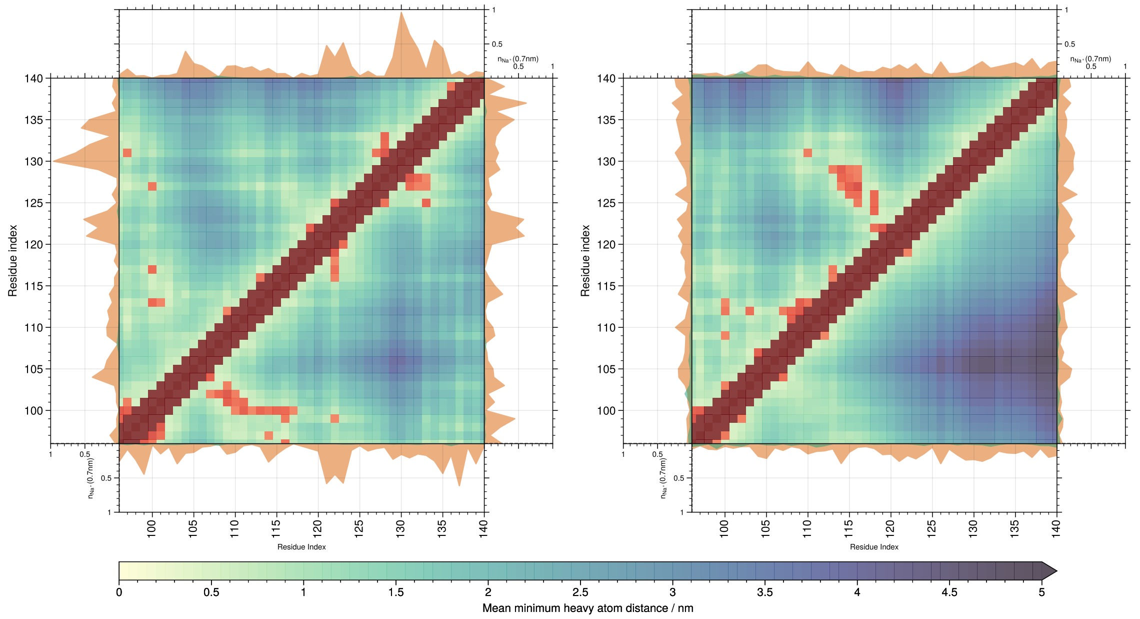

Figure S7: Distance maps between residues 96-140 in representative compact (triangle above) and extended (triangle below) clusters in wild-type (left) and E46K mutated (right) AS. Red points mark contact (mean distance $\leq$ 0.5 nm). Insets show the mean number of sodium (orange) and chloride (green) ions within a distance of 0.70 nm.

4.6 *Sodium Distribution Across Clusters and Mutation*

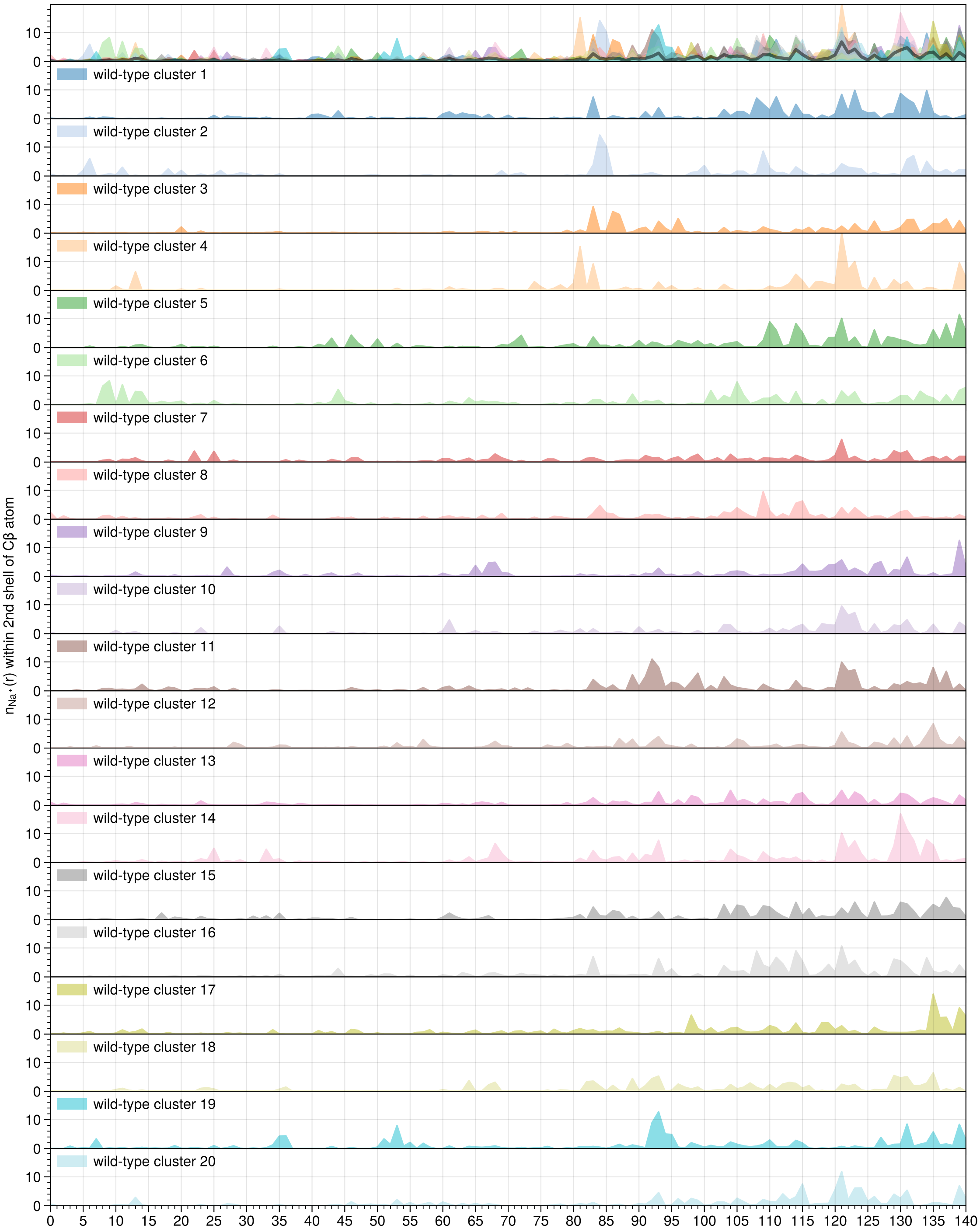

Figure S8: Distribution of sodium ions across different clusters of wild-type AS. Values are the mean number of sodium ions up to 0.70 nm from the ion interaction sites in each residue.

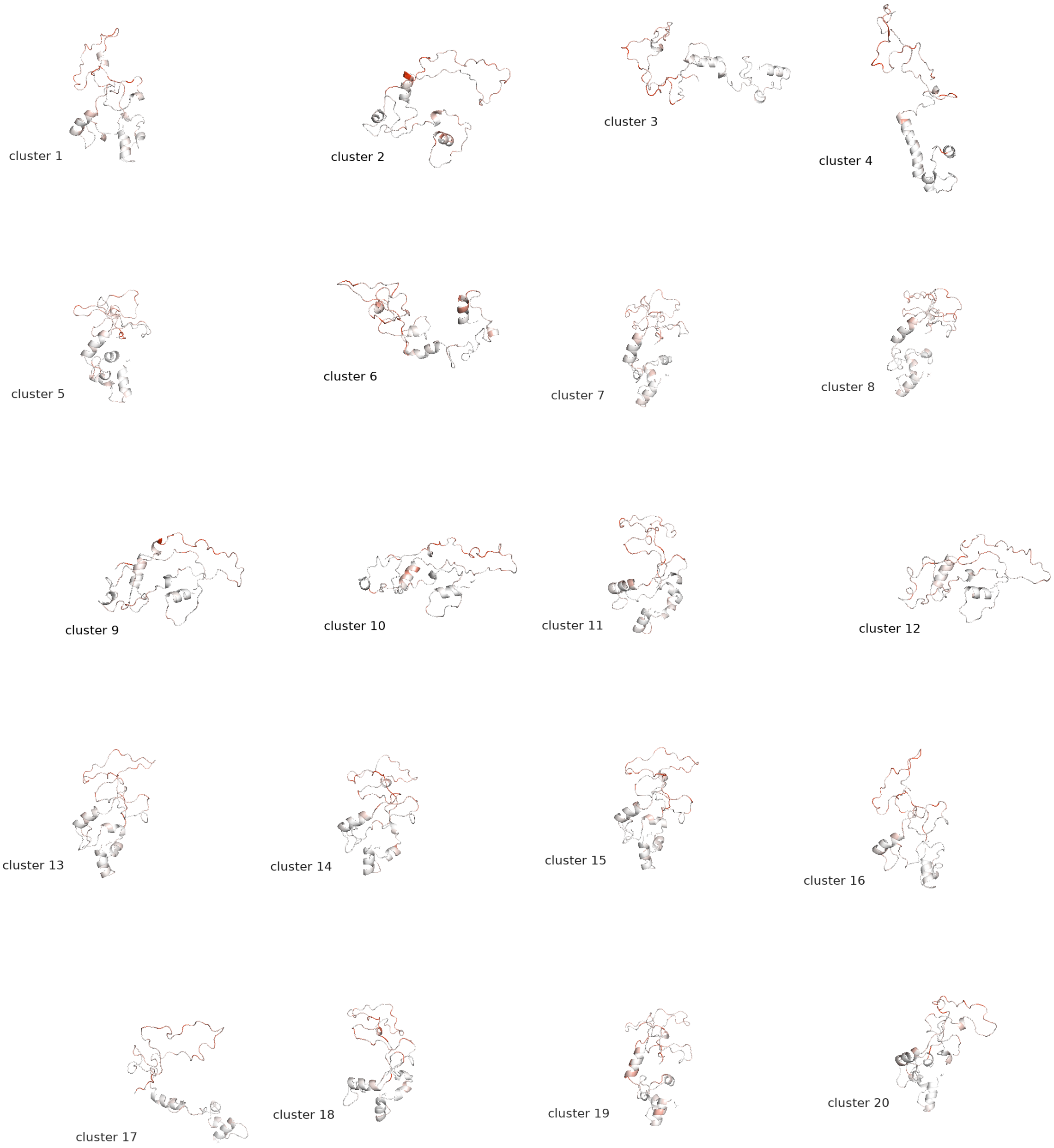

Figure S9: Visualization of the distribution of sodium ions present within the first and second coordination shells of the ion interaction sites in conformational clusters of wild-type alpha-Synuclein. Red coloring indicates increasing $n(r)$.

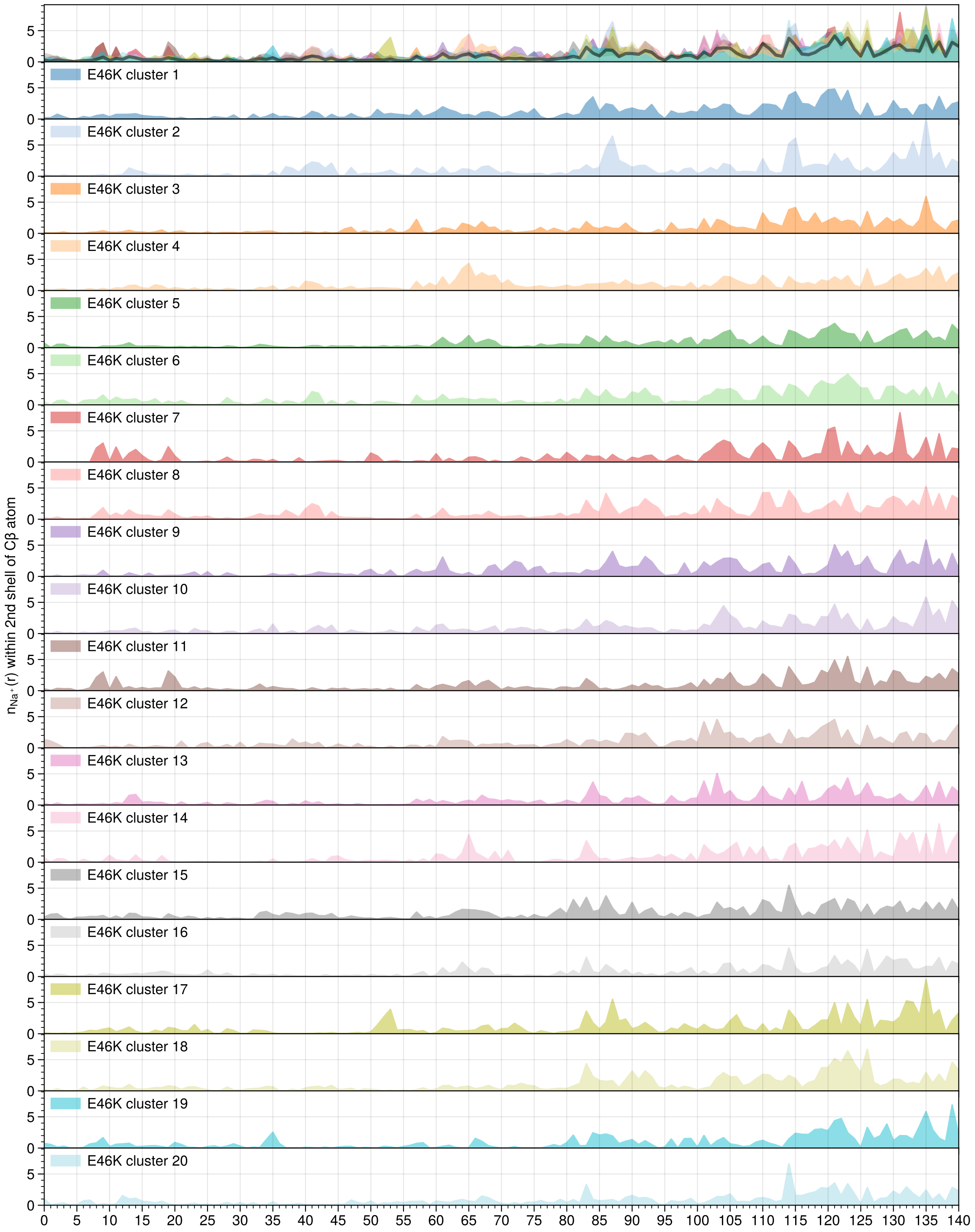

Figure S10: Distribution of sodium ions across different clusters of E46K-AS. Values are the mean number of sodium ions up to 0.70 nm from the ion interaction sites in each residue.

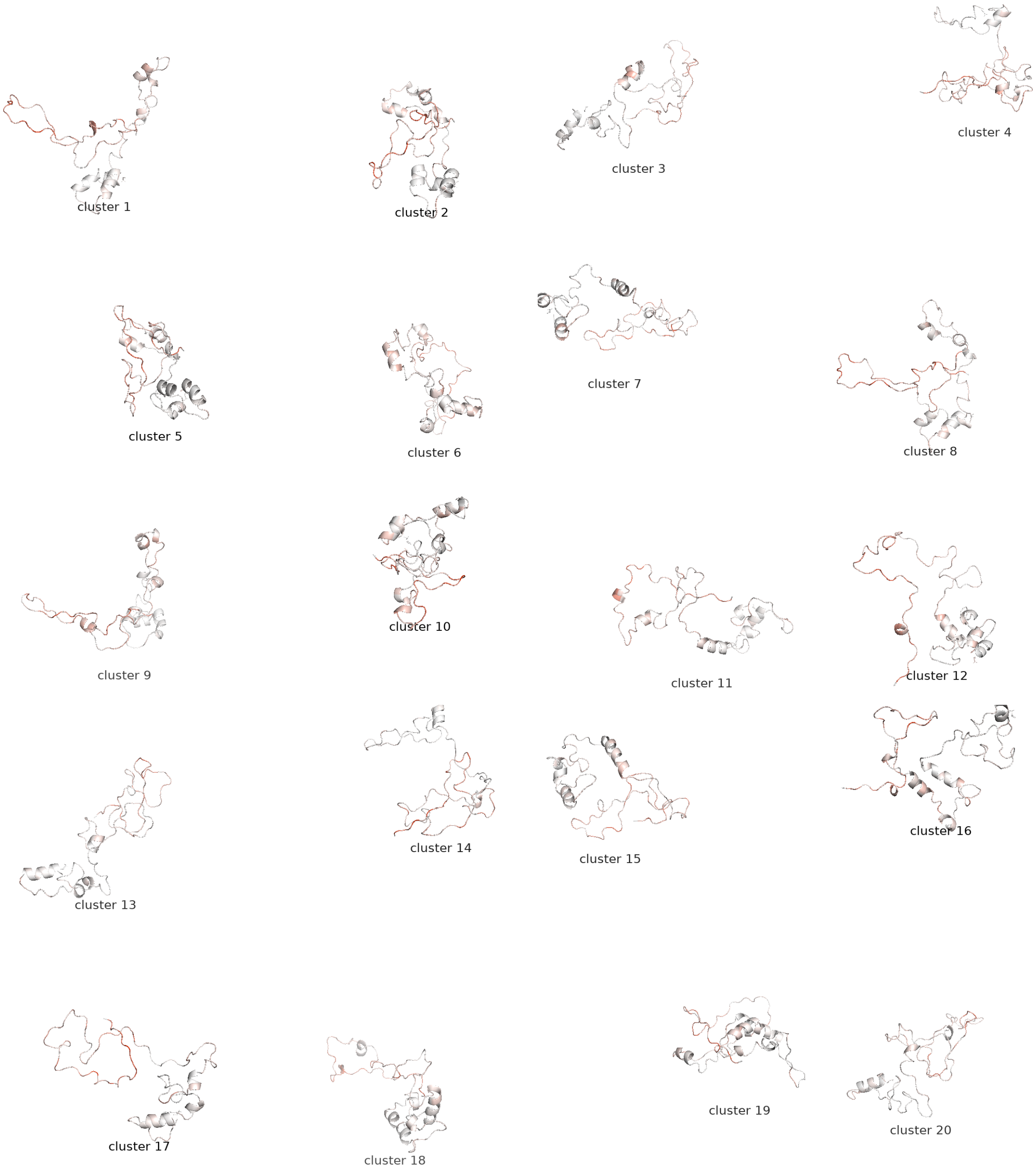

Figure S11: Visualization of the distribution of sodium ions present within the first and second coordination shells of the ion interaction sites atoms in conformational clusters of E46K mutated alpha-Synuclein. Red coloring indicates increasing $n(r)$.

4.7 *Chloride Distribution Across Clusters and Mutation*

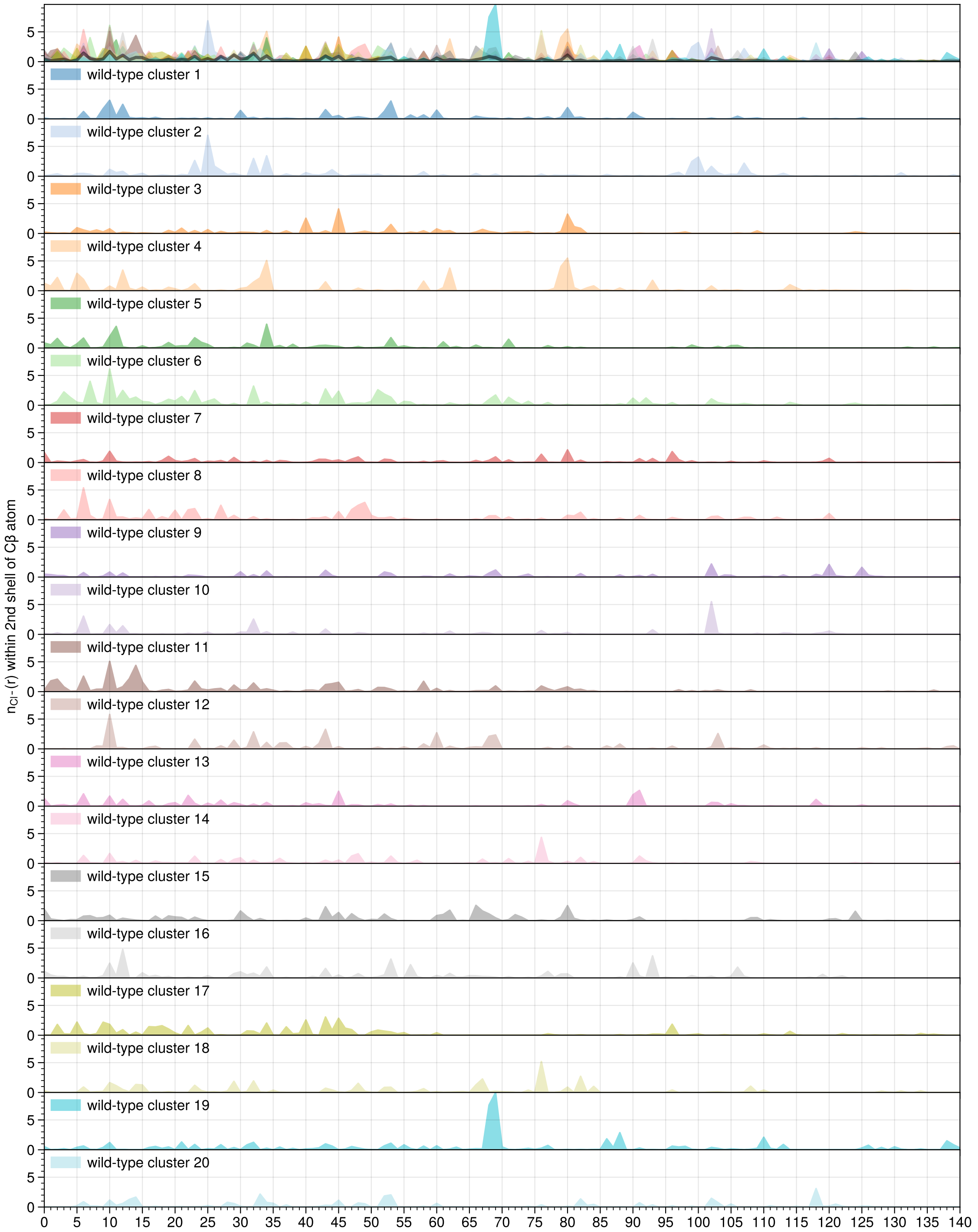

Figure S12: Distribution of chloride ions across different clusters of wild-type AS. Values are the mean number of chloride ions up to 0.70 nm from the ion interaction sites in each residue.

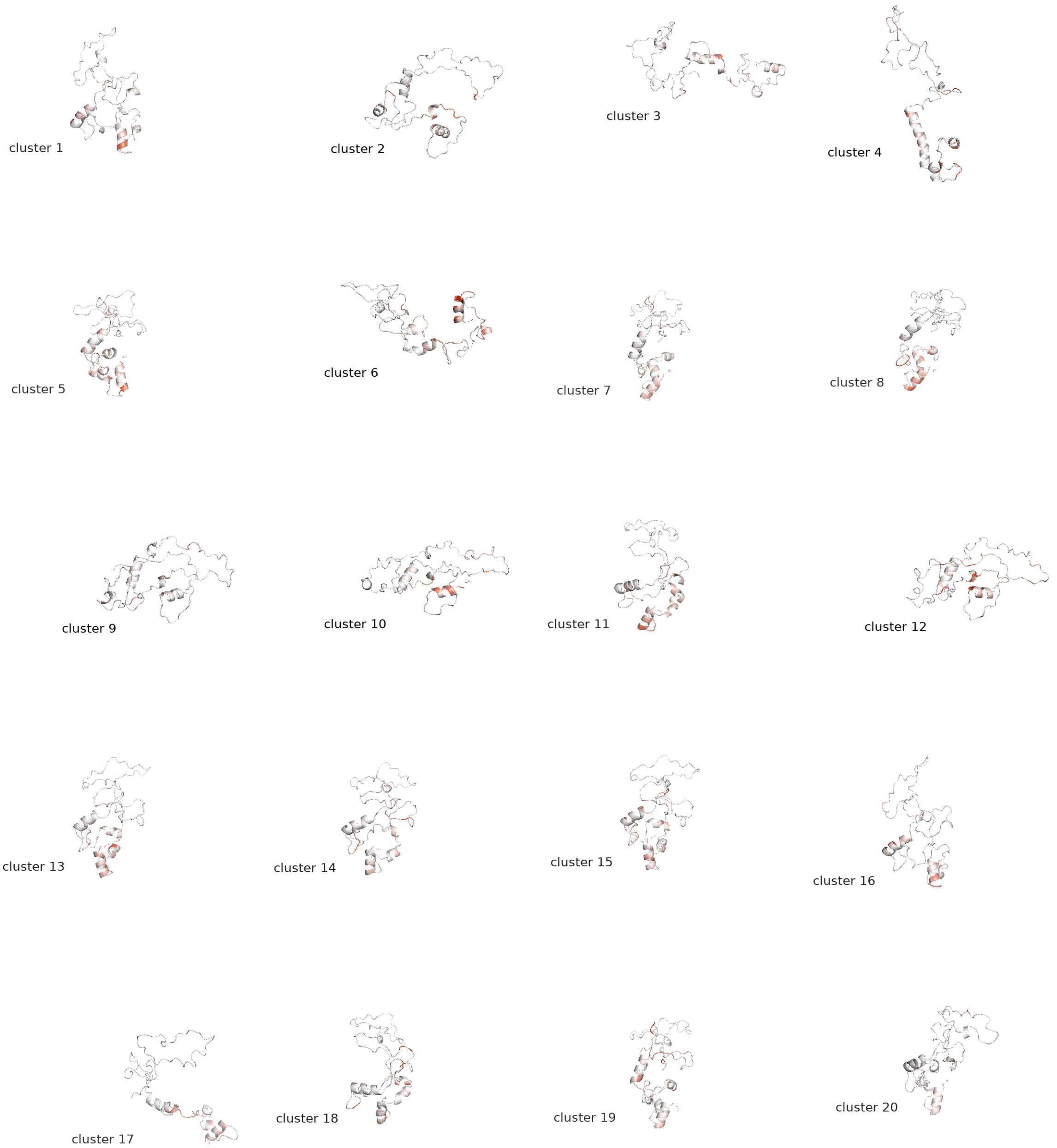

Figure S13: Visualization of the distribution of chloride ions present within the first and second coordination shells of the ion interaction sites in conformational clusters of wild-type alpha-Synuclein. Red coloring indicates increasing $n(r)$.

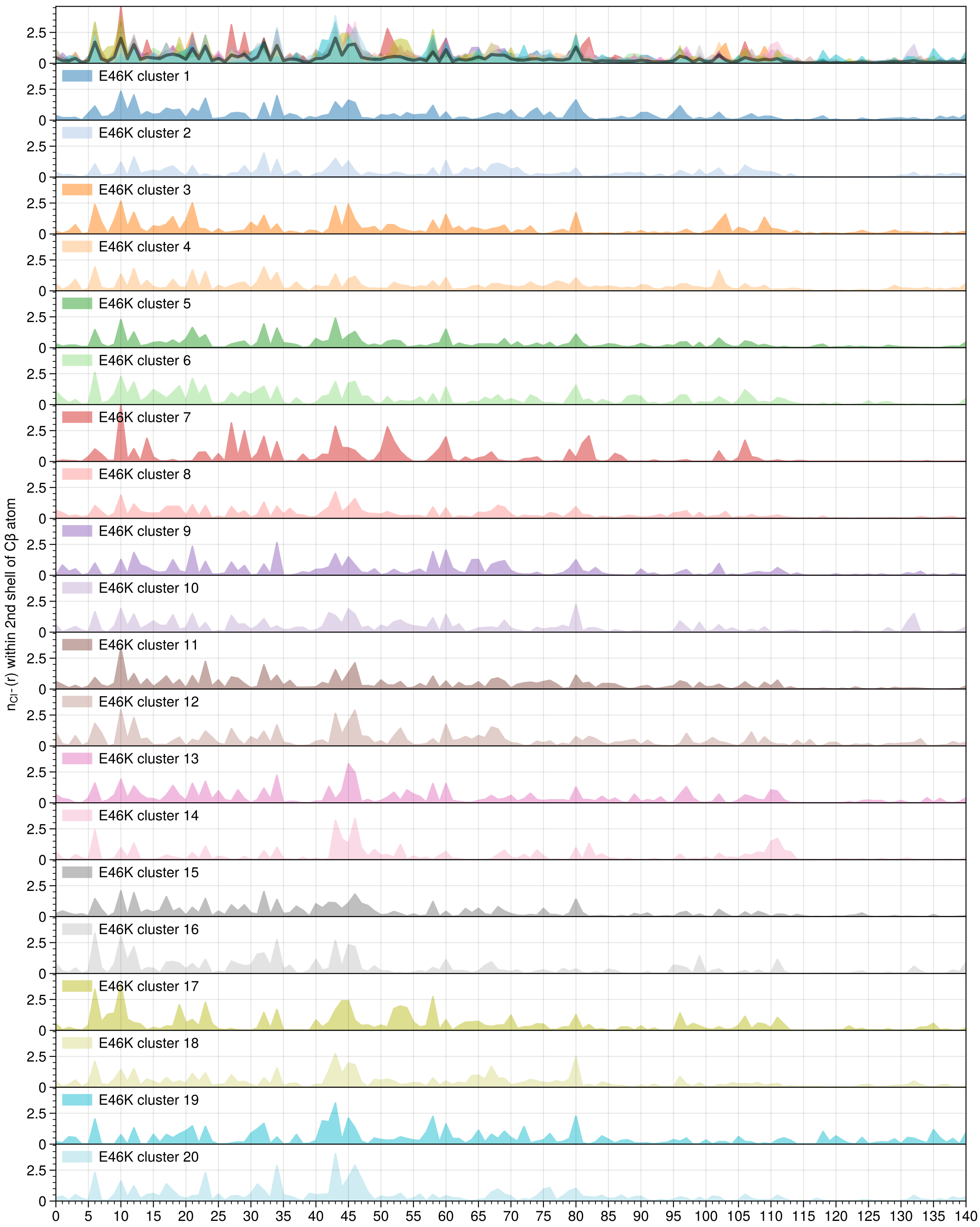

Figure S14: Distribution of chloride ions across different clusters of E46K-AS. Values are the mean number of chloride ions up to 0.70 nm from the ion interaction sites in each residue.

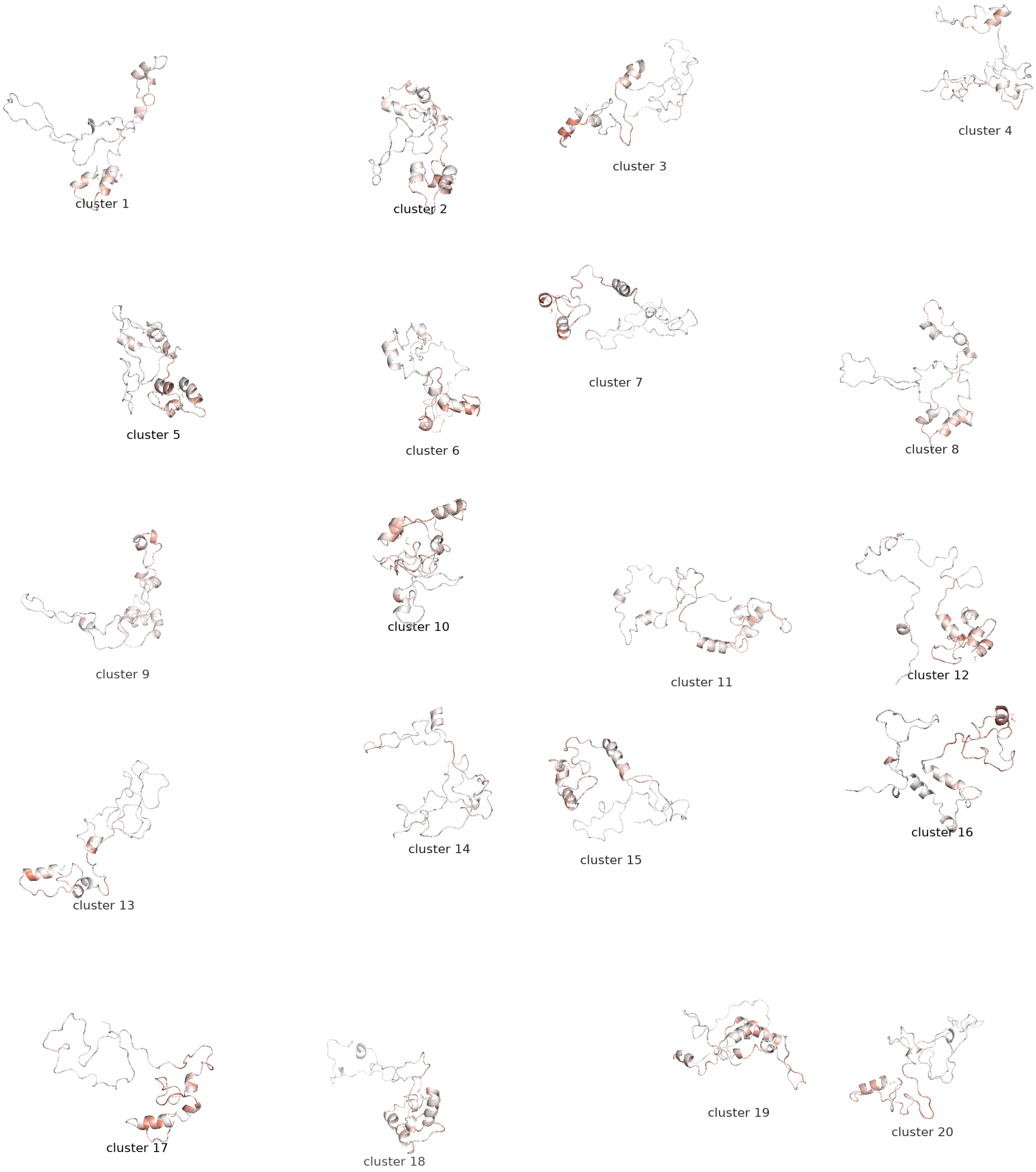

Figure S15: Visualization of the distribution of chloride ions present within the first and second coordination shells of the ion interaction sites in conformational clusters of E46K mutated alpha-Synuclein. Red coloring indicates increasing $n(r)$.

5 COMPARISON OF FORCE FIELDS USING WILD-TYPE ALPHA-SYNUCLEIN

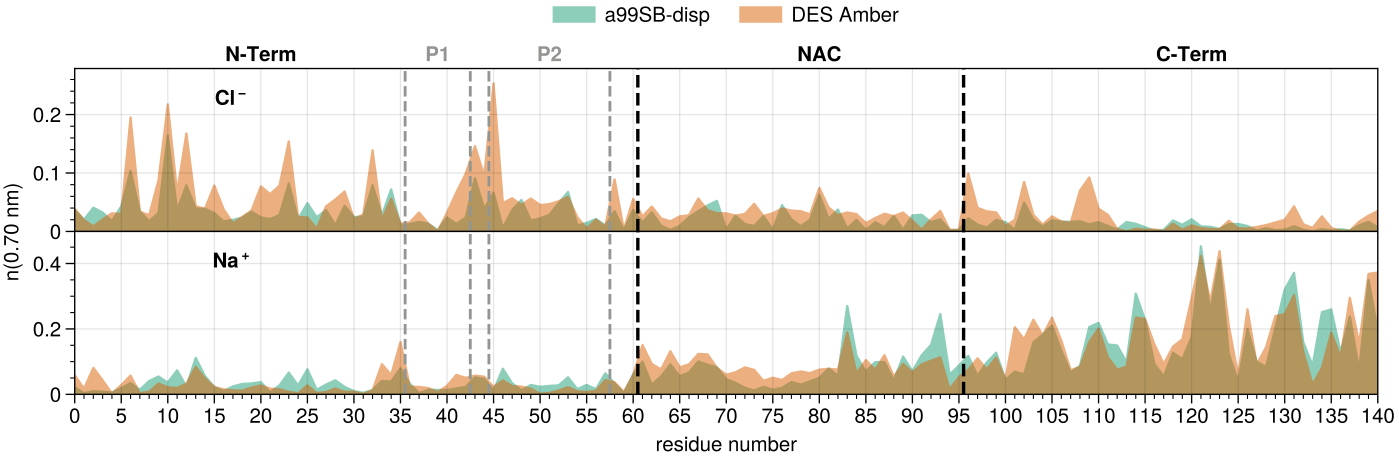

Figure S16: Distribution of ions around alpha-Synuclein (AS) during simulations using different force field parameters. Color intensity scales with difference between the running average $n(r,t)$ and total average $n(r)$. The presence of ions was averaged over 13 ns of simulation time, after 12 ns of equilibration.

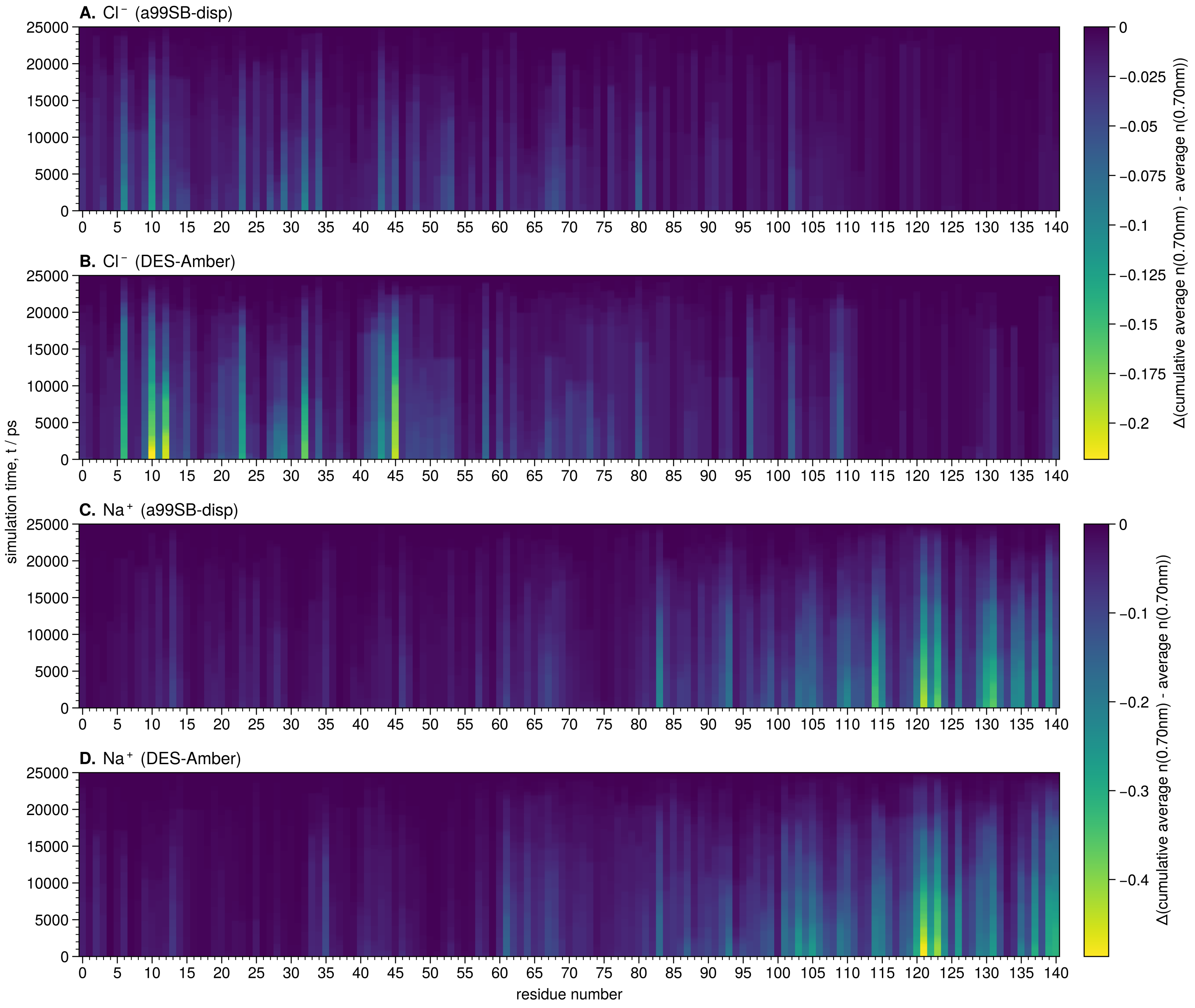

Figure S17: Equilibration of ions around AS during 25 ns of simulation time using different force field parameters. Color intensity shows the difference between cumulative ion occupation and mean ion occupation.

6 HUMANIN

6.1 *Ion Distribution At Large Distance*

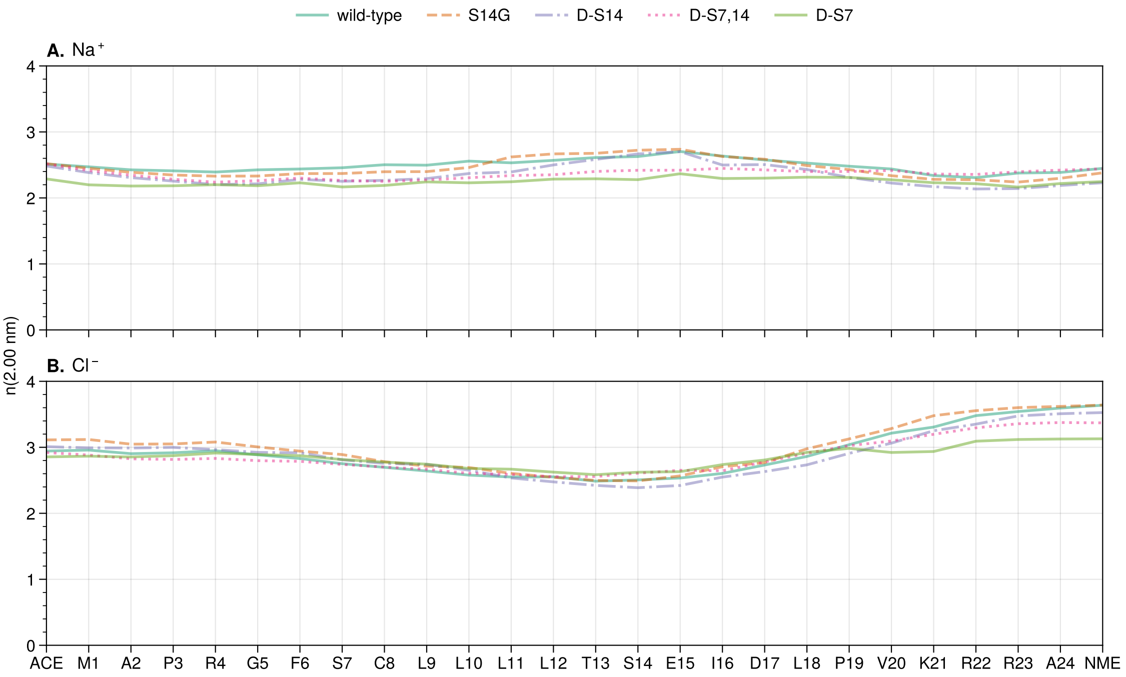

Figure S18: Sodium (A) and Chloride (B) ion distribution along the residue index for wild-type and mutants of Humanin (HN). The mean number of ions is given as the Radial Distribution Function (RDF) around each functional site integrated up to 2.00 nm.

6.2 *Comparison of Equilibration Between Unbiased and REST2 MD*

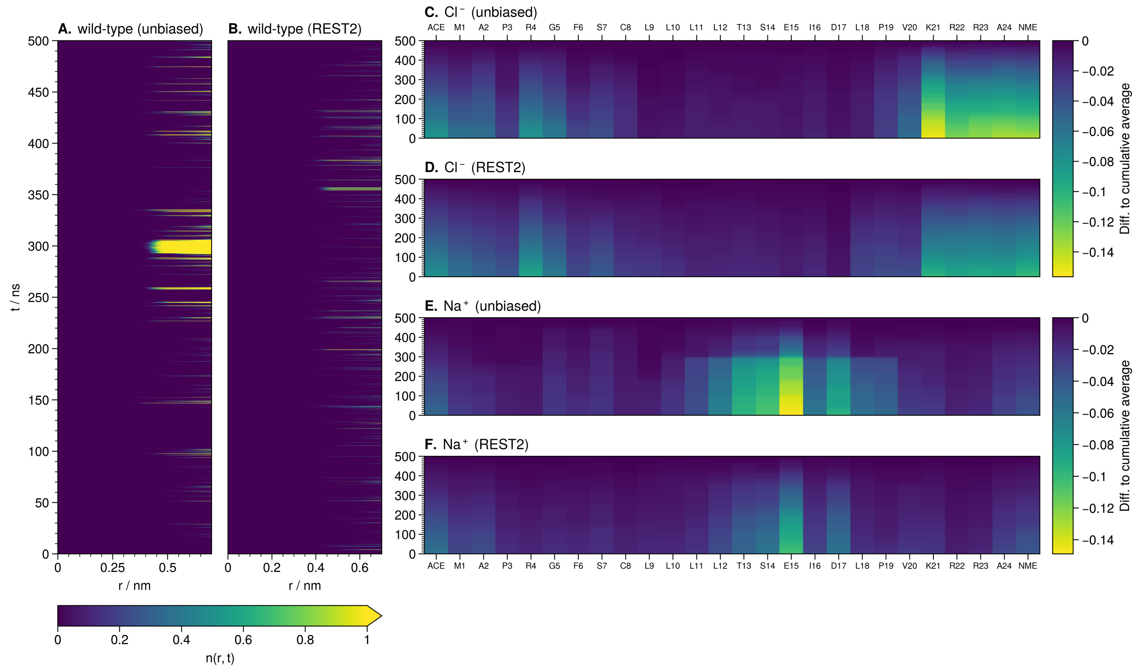

Figure S19: Distribution of ions around HN during simulations using biased and REST2 enhanced sampling Molecular Dynamics (MD) algorithms. The force field and starting structures were kept the same. Colors intensity scales with difference between the running average $n(r,t)$ and total average $n(r)$. Ion concentrations were averaged over the first 500 ns of simulation time.

6.3 *Van Hove Function Applied to the S14 Residue in Humanin*

The dynamics of sodium ions in the vicinity of S14 can be further explored by constructing van Hove Functions (VHFs) of different time slices along the trajectory. The VHF in figure S20B shows that a single sodium ion near S14 stays in the vicinity of the residue, and because of the well defined peaks, there seems to be little or no movement of the ion relative to the atoms in the residue itself. The VHF in figure S20C shows an absence or very low likelihood of finding a sodium ion near S14 during parts of the simulation. Some information can still be gained from this however, as the lack of decline in VHF along displacement time again shows a stable broad peak, meaning that the sodium ions at the distance of the broad peak are not moving rapidly with respect to the residue. Figure S20A shows a part of the trajectory corresponding to another peak in the Time-Resolved Radial Distribution Function (TRRDF). This shows that at least one sodium ion is near S14 at a consistent distance during this part of the simulation, and simultaneously that there is significant lateral movement with respect to the single atoms in the residue.

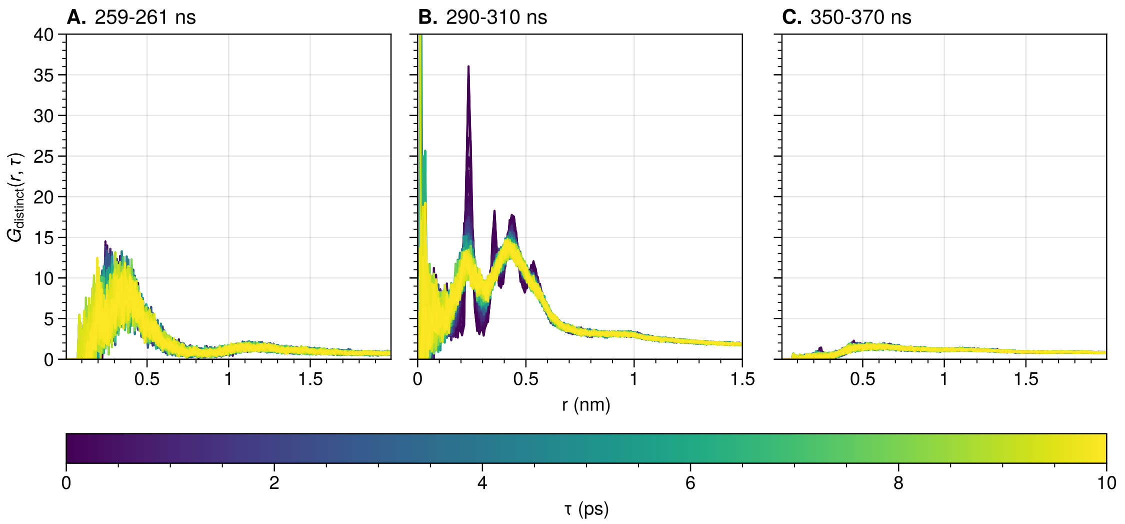

Figure S20: van Hove Functions (VHFs) for sodium at S14 with respect to sodium ions at three different simulation times: 259-261 ns (A), 290-310 ns (B) and 350-370 ns (C). Window length: 10 ps.

The VHFs distinguish between different oscillations and movements that cannot be seen using only time-averaged RDFs. Examining the decay of certain peaks in the VHF also allows the quantification of phenomena that might otherwise only be qualitatively assessed using visual inspection of the simulation trajectory.

7 PERFORMANCE BENCHMARK AND COMPARISON TO OTHER PACKAGES

7.1 *Distance Evaluations With The Minimum Image Convention*

The most computational intensive part of calculating radial distribution functions is obtaining the distance matrix between particles. For N_{target} target particles together with M_{ref} reference particles, a total of $3N_{\text{target}} \times M_{\text{reference}}$ distances need to be evaluated over 3 cartesian coordinates. As Molecular Dynamics (MD) simulations are generally conducted using reduced-size *periodic* simulation cells, correct distances between particles have to be calculated using the Minimum Image Convention (MIC). In general, each component distance along a geometry axis Δx_0 between particle n and particle m_0 inside the simulation cell are compared to the distances Δx_{-1} and Δx_{+1} to the periodic image of the reference particle (m_{-1} and m_{+1}). An orthogonal simulation cell has 3 axes and is generally the least computationally intensive for analysis. The highest computational complexity is found in dodecahedral simulation cells, where a total of 9 axes are present and where each inter-cell distance is compared to 26 mirror image distances.

SPEADI implements an efficient distance-matrix algorithm[29] that calculates MIC distances either for an orthogonal geometry or for an arbitrary geometry. Distances for arbitrary geometries incur a runtime penalty of 2 to 5. Figure S21 shows a benchmark comparison between SPEADI and the distance-matrix function implemented in the MDTraj Python package. Both algorithms are implemented using shared-memory parallelism.

Table S2: Execution time in seconds over a single trajectory window for generating simultaneous radial distribution functions between 1, 10, and 100 single carbon atoms and 1000 and 10000 solvent atoms. Each window consists of 10 trajectory frames.

**System configuration**	**N(distances)** / 10^3	**MDAnalysis**	**SPEADI (jax)**	**SPEADI (non-opt)**	**SPEADI (numba)**	**MDTraj**
2 × Intel(R) Xeon(R) CPU 2.20 GHz	10	1.159	1.043	0.996	0.950	1.281
	100	1.163	1.073	1.121	0.980	1.307
	1000	1.958	1.047	2.735	1.278	1.609
	10000	10.538	1.228	6.185	4.321	4.179
256 × AMD EPYC 7742 64-Core Processor	10	0.251	0.209	0.215	0.212	0.278
	100	0.271	0.217	0.255	0.230	0.299
	1000	0.802	0.232	0.673	0.399	0.604
	10000	10.346	0.364	3.389	2.081	3.204
8 × Apple M1	10	1.965	1.501	1.537	1.544	2.090
	100	2.073	1.597	1.618	1.524	2.424
	1000	2.387	1.534	2.030	1.686	2.302
	10000	7.183	1.631	4.835	3.177	4.218
80 × Intel(R) Xeon(R) Gold 6148 CPU 2.40 GHz	10	0.304	0.269	0.275	0.271	0.351
	100	0.340	0.270	0.311	0.297	0.366
	1000	0.855	0.282	0.688	0.545	0.563
	10000	8.296	0.294	2.422	3.026	2.136

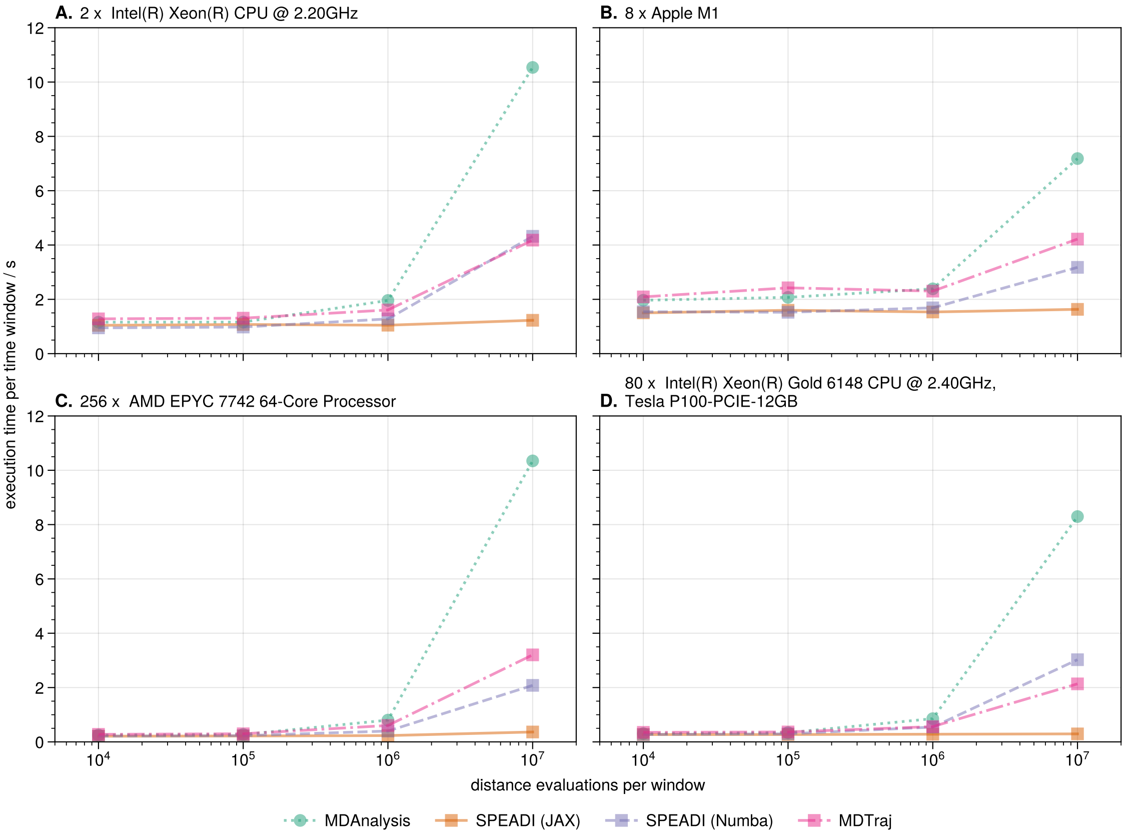

Figure S21: Comparison of execution time per window calculated and scaling according to the number of distance evaluations benchmarked on various hardware. The benchmarks in (A–C) were run using only CPUs. The benchmark in (D) was run with an Nvidia Tesla P100 GPU available to the functions.

REFERENCES

[1] Yuya Shinohara, Ray Matsumoto, Matthew W. Thompson, Chae Woo Ryu, Wojciech Dmowski, Takuya Iwashita, Daisuke Ishikawa, Alfred Q. R. Baron, Peter T. Cummings, and Takeshi Egami. Identifying water-anion correlated motion in aqueous solutions through van hove functions. *The Journal of Physical Chemistry Letters*, 10(22):7119–7125, 2019. doi: 10.1021/acs.jpclett.9b02891. URL https://doi.org/10.1021/acs.jpclett.9b02891.

[2] Yuya Shinohara, Wojciech Dmowski, Takuya Iwashita, Daisuke Ishikawa, Alfred Q. R. Baron, and Takeshi Egami. Local correlated motions in aqueous solution of sodium chloride. *Physical Review Materials*, 3(6):065604, 2019. doi: 10.1103/physrevmaterials.3.065604. URL https://doi.org/10.1103/physrevmaterials.3.065604.

[3] Takeshi Egami and Yuya Shinohara. Correlated atomic dynamics in liquid seen in real space and time. *The Journal of Chemical Physics*, 153(18):180902, 2020. doi: 10.1063/5.0024013. URL https://doi.org/10.1063/5.0024013.

[4] Léon van Hove. Correlations in space and time and born approximation scattering in systems of interacting particles. *Physical Review*, 95(1):249–262, 1954. doi: 10.1103/physrev.95.249.

URL https://doi.org/10.1103/physrev.95.249.

[5] I. R. McDonald Jean-Pierre Hansen. *Theory of Simple Liquids*. Elsevier Science & Techn, 2006. URL https://www.ebook.de/de/product/15247290/jean_pierre_hansen_i_r_mcdonald_theory_of_simple_liquids.html.

[6] Paul Hopkins, Andrea Fortini, Andrew J. Archer, and Matthias Schmidt. The van hove distribution function for brownian hard spheres: Dynamical test particle theory and computer simulations for bulk dynamics. *The Journal of Chemical Physics*, 133(22):224505, 2010. doi: 10.1063/1.3511719. URL https://doi.org/10.1063/1.3511719.

[7] Zahra Ghannad. Fickian yet non-gaussian diffusion in two-dimensional yukawa liquids. *Physical Review E*, 100(3):033211, 2019. doi: 10.1103/physreve.100.033211. URL http://dx.doi.org/10.1103/PhysRevE.100.033211.

[8] M. Paul Lettinga, Laura Alvarez, Olivera Korculanin, and Eric Grelet. When bigger is faster: a self-van hove analysis of the enhanced self-diffusion of non-commensurate guest particles in smectics. *The Journal of Chemical Physics*, 154(20):204901, 2021. doi: 10.1063/5.0049093. URL http://dx.doi.org/10.1063/5.0049093.

[9] Robert T. McGibbon, Kyle A. Beauchamp, Matthew P. Harrigan, Christoph Klein, Jason M. Swails, Carlos X. Hernández, Christian R. Schwantes, Lee-Ping Wang, Thomas J. Lane, and Vijay S. Pande. Mdtraj: a modern open library for the analysis of molecular dynamics trajectories. *Biophysical Journal*, 109(8):1528–1532, 2015. doi: 10.1016/j.bpj.2015.08.015. URL https://doi.org/10.1016/j.bpj.2015.08.015.

[10] James Bradbury, Roy Frostig, Peter Hawkins, Matthew James Johnson, Chris Leary, Dougal Maclaurin, George Necula, Adam Paszke, Jake VanderPlas, Skye Wanderman-Milne, and Qiao Zhang. JAX: composable transformations of Python+NumPy programs (v0.2.5), 2018. URL http://github.com/google/jax.

[11] Andrew W. Senior, Richard Evans, John Jumper, James Kirkpatrick, Laurent Sifre, Tim Green, Chongli Qin, Augustin Žídek, Alexander W. R. Nelson, Alex Bridgland, Hugo Penedones, Stig Petersen, Karen Simonyan, Steve Crossan, Pushmeet Kohli, David T. Jones, David Silver, Koray Kavukcuoglu, and Demis Hassabis. Improved protein structure prediction using potentials from deep learning. *Nature*, 577(7792):706–710, 2020. doi: 10.1038/s41586-019-1923-7. URL http://dx.doi.org/10.1038/s41586-019-1923-7.

[12] John Jumper, Richard Evans, Alexander Pritzel, Tim Green, Michael Figurnov, Olaf Ronneberger, Kathryn Tunyasuvunakool, Russ Bates, Augustin Žídek, Anna Potapenko, Alex Bridgland, Clemens Meyer, Simon A. A. Kohl, Andrew J. Ballard, Andrew Cowie, Bernardino Romera-Paredes, Stanislav Nikolov, Rishub Jain, Jonas Adler, Trevor Back, Stig Petersen, David Reiman, Ellen Clancy, Michal Zielinski, Martin Steinegger, Michalina Pacholska, Tamas Berghammer, Sebastian Bodenstein, David Silver, Oriol Vinyals, Andrew W. Senior, Koray Kavukcuoglu, Pushmeet Kohli, and Demis Hassabis. Highly accurate protein structure prediction with alphafold. *Nature*, 596(7873):583–589, 2021. doi: 10.1038/s41586-021-03819-2. URL http://dx.doi.org/10.1038/s41586-021-03819-2.

[13] Martín Abadi, Ashish Agarwal, Paul Barham, Eugene Brevdo, Zhifeng Chen, Craig Citro, Greg S. Corrado, Andy Davis, Jeffrey Dean, Matthieu Devin, Sanjay Ghemawat, Ian Goodfellow, Andrew Harp, Geoffrey Irving, Michael Isard, Yangqing Jia, Rafal Jozefowicz, Lukasz Kaiser, Manjunath Kudlur, Josh Levenberg, Dandelion Mané, Rajat Monga, Sherry Moore,

Derek Murray, Chris Olah, Mike Schuster, Jonathon Shlens, Benoit Steiner, Ilya Sutskever, Kunal Talwar, Paul Tucker, Vincent Vanhoucke, Vijay Vasudevan, Fernanda Viégas, Oriol Vinyals, Pete Warden, Martin Wattenberg, Martin Wicke, Yuan Yu, and Xiaoqiang Zheng. TensorFlow: Large-scale machine learning on heterogeneous systems, 2015. URL https://www.tensorflow.org/. Software available from tensorflow.org.

[14] Siu Kwan Lam, Antoine Pitrou, and Stanley Seibert. Numba. In *Proceedings of the Second Workshop on the LLVM Compiler Infrastructure in HPC - LLVM '15*, page nil, - 2015. doi: 10.1145/2833157.2833162. URL https://doi.org/10.1145/2833157.2833162.

[15] Stéfan van der Walt, S Chris Colbert, and Gaël Varoquaux. The numpy array: a structure for efficient numerical computation. *Computing in Science & Engineering*, 13(2):22–30, 2011. doi: 10.1109/mcse.2011.37. URL https://doi.org/10.1109/mcse.2011.37.

[16] Charles R. Harris, K. Jarrod Millman, Stéfan J. van der Walt, Ralf Gommers, Pauli Virtanen, David Cournapeau, Eric Wieser, Julian Taylor, Sebastian Berg, Nathaniel J. Smith, Robert Kern, Matti Picus, Stephan Hoyer, Marten H. van Kerkwijk, Matthew Brett, Allan Haldane, Jaime Fernández del Río, Mark Wiebe, Pearu Peterson, Pierre Gérard-Marchant, Kevin Sheppard, Tyler Reddy, Warren Weckesser, Hameer Abbasi, Christoph Gohlke, and Travis E. Oliphant. Array programming with numpy. *Nature*, 585(7825):357–362, 2020. doi: 10.1038/s41586-020-2649-2. URL https://doi.org/10.1038/s41586-020-2649-2.

[17] Lingle Wang, Richard A. Friesner, and B. J. Berne. Replica exchange with solute scaling: a more efficient version of replica exchange with solute tempering (rest2). *The Journal of Physical Chemistry B*, 115(30):9431–9438, 2011. doi: 10.1021/jp204407d. URL https://doi.org/10.1021/jp204407d.

[18] G. Rossetti, F. Musiani, E. Abad, D. Dibenedetto, H. Mouhib, C. O. Fernandez, and P. Carloni. Conformational ensemble of human α-synuclein physiological form predicted by molecular simulations. *Physical Chemistry Chemical Physics*, 18(8):5702–5706, 2016. doi: 10.1039/c5cp04549e. URL https://doi.org/10.1039/c5cp04549e.

[19] Paul Robustelli, Stefano Piana, and David E. Shaw. Developing a molecular dynamics force field for both folded and disordered protein states. *Proceedings of the National Academy of Sciences*, 115(21):E4758–E4766, 2018. doi: 10.1073/pnas.1800690115. URL https://doi.org/10.1073/pnas.1800690115.

[20] Dimitra Benaki, Christos Zikos, Alexandra Evangelou, Evangelia Livaniou, Metaxia Vlassi, Emmanuel Mikros, and Maria Pelecanou. Solution structure of humanin, a peptide against alzheimer's disease-related neurotoxicity. *Biochemical and Biophysical Research Communications*, 329(1):152–160, 2005. doi: 10.1016/j.bbrc.2005.01.100. URL https://doi.org/10.1016/j.bbrc.2005.01.100.

[21] LLC Schrödinger and Warren DeLano. Pymol. URL http://www.pymol.org/pymol.

[22] H.J.C. Berendsen, D. van der Spoel, and R. van Drunen. Gromacs: a message-passing parallel molecular dynamics implementation. *Computer Physics Communications*, 91(1-3):43–56, 1995. doi: 10.1016/0010-4655(95)00042-e. URL http://dx.doi.org/10.1016/0010-4655(95)00042-E.

[23] Erik Lindahl, Berk Hess, and David van der Spoel. Gromacs 3.0: a package for molecular simulation and trajectory analysis. *Journal of Molecular Modeling*, 7(8):306–317, 2001. doi: 10.1007/s008940100045. URL http://dx.doi.org/10.1007/s008940100045.

[24] David Van Der Spoel, Erik Lindahl, Berk Hess, Gerrit Groenhof, Alan E. Mark, and Herman J. C. Berendsen. Gromacs: Fast, flexible, and free. *Journal of Computational Chemistry*, 26(16): 1701–1718, 2005. doi: 10.1002/jcc.20291. URL http://dx.doi.org/10.1002/jcc.20291.

[25] Berk Hess, Carsten Kutzner, David van der Spoel, and Erik Lindahl. Gromacs 4: Algorithms for highly efficient, load-balanced, and scalable molecular simulation. *Journal of Chemical Theory and Computation*, 4(3):435–447, 2008. doi: 10.1021/ct700301q. URL http://dx.doi.org/10.1021/ct700301q.

[26] Sander Pronk, Szilárd Páll, Roland Schulz, Per Larsson, Pär Bjelkmar, Rossen Apostolov, Michael R. Shirts, Jeremy C. Smith, Peter M. Kasson, David van der Spoel, Berk Hess, and Erik Lindahl. Gromacs 4.5: a high-throughput and highly parallel open source molecular simulation toolkit. *Bioinformatics*, 29(7):845–854, 2013. doi: 10.1093/bioinformatics/btt055. URL http://dx.doi.org/10.1093/bioinformatics/btt055.

[27] Mark James Abraham, Teemu Murtola, Roland Schulz, Szilárd Páll, Jeremy C. Smith, Berk Hess, and Erik Lindahl. Gromacs: High performance molecular simulations through multi-level parallelism from laptops to supercomputers. *SoftwareX*, 1-2(nil):19–25, 2015. doi: 10.1016/j.softx.2015.06.001. URL http://dx.doi.org/10.1016/j.softx.2015.06.001.

[28] Rajeswari Appadurai, Jaya Krishna Koneru, Massimiliano Bonomi, Paul Robustelli, and Anand Srivastava. Demultiplexing the heterogeneous conformational ensembles of intrinsically disordered proteins into structurally similar clusters, 2022. URL http://dx.doi.org/10.1101/2022.11.11.516231.

[29] Mark Tuckerman. *Statistical mechanics: theory and molecular simulation*. Oxford University Press, Oxford, 2010. ISBN 9780198525264.
